# Molecular characterization of pulmonary defenses against bacterial invasion in allergic asthma: The role of Foxa2 in regulation of β-defensin 1

**DOI:** 10.1371/journal.pone.0226517

**Published:** 2019-12-27

**Authors:** Chuanqi Wei, Xiaoju Tang, Faping Wang, Yan Li, Lin Sun, Fengming Luo

**Affiliations:** Department of Pulmonary and Critical Care Medicine, West China Hospital, Sichuan University, Guo Xue Xiang, Chengdu, China; Albany Medical College, UNITED STATES

## Abstract

Allergic asthma, characterized by chronic airway Th2-dominated inflammation, is associated with an increased risk of infection; however, the underlying mechanisms are unclear. Forkhead box protein A2 (Foxa2) plays a critical role in Th2 inflammation and is associated with pulmonary defenses. To determining the role of Foxa2 in Th2-dominated lung inflammation against the invading bacteria, we established a mouse OVA-sensitized model, an *Escherichia coli* lung invasion model, and mice with conditional deletion of Foxa2 in respiratory epithelial cells. The number of bacteria in the lung tissue was counted to assess clearance ability of lung. Lung inflammation and histopathology was evaluated using HE and PAS staining. It was found that OVA-sensitized mice had decreased *E*. *coli* clearance, reduced Foxa2 expression, and decreased DEFB1 secretion. Conditional deletion of Foxa2 in respiratory epithelial cells led to decreased clearance of *E*. *coli* and impaired secretion of DEFB1, similar to the OVA-induced allergic condition. The impaired secretion of DEFB1 may be responsible for the increased risk of infection in the Th2-dominated airway inflammation. Dual luciferase assay demonstrated that Foxa2 regulates DEFB1 expression by affecting its promoter activity in HBE cells. Our study indicated that Foxa2 plays an important role in Th2-dominated airway inflammation against invading bacteria. Conditional deletion of Foxa2 in respiratory epithelial cells can reduce pulmonary’s defense against bacterial invasion by inhibiting DEFB1expression.

## Introduction

Asthma is a common disease clinically characterized by chronic T helper type 2 (Th2)-dominated airway inflammation that affects approximately 1–18% of the population globally [1]. Inhaled allergens play a vital role in the pathogenesis and development of asthma; hence, asthma is often categorized as an atopic condition [1–3]. Increased prevalence surveys and studies from animals showed that asthma increases the risk of infections in the host [3–7]. The predominant infections include respiratory tract infections, skin infections, urinary tract infections, and sepsis [3–5]. Currently, the mechanisms underlying the increased risk of infections are not fully understood. Elucidation of the mechanisms of pulmonary defense against microorganisms may help improve the management and prevention of infections in patients with asthma. For the invading microbes, the first barrier is the airway epithelial cells lining the respiratory tract and the antimicrobial substances produced by these cells [8]. Among these, β-defensins (DEFBs), located in a β-defensin gene cluster on chromosome band 8p.23.1 [9], are critical for mediating innate immunity and host defenses [8,10]. DEFBs are small endogenous peptides that possess a wide range of microbicidal activity against bacteria, some fungi, and enveloped viruses [8,10]. Earlier studies have shown persistent bacterial infection and the inability to clear bacteria from the epithelial surface in patients with CF, which is characterized by chronic airway infection and impaired expression of DEFBs [10,11]. Among DEFBs, β-defensin 1(DEFB1) is an essential element of innate mucosal immunity and is expressed constitutively by the epithelial cells [11]. Studies on DEFB1-deficient mice showed compromised innate immunity and increased infection risk; however, adaptive immunity was functional when DEFB1 was mutated [12,13]. DEFB1 was shown to be involved in the induction of other DEFBs, such as DEFB2, DEFB3, and DEFB4 [12,14]. Earlier studies have elucidated an association between DEFB1 polymorphisms and the pathogenesis of asthma [15,16]. Some observational studies also reported diverse but conflicting expression levels of DEFB1 in mouse asthma models and clinical trials [6,17]. However, the role of DEFB1 in the development and pathogenesis of asthma is unclear. The transcription factor forkhead box protein A2 (Foxa2, also known as hepatocyte nuclear factor 3-beta [HNF-3B], or transcription factor 3B [TCF-3B]) plays an essential role in regulating Th2 inflammation and airway mucus secretion in the developing lung [18]. Deletion of the Foxa2 gene in respiratory epithelial cells of the mouse leads to spontaneous pulmonary eosinophilic inflammation, goblet cell metaplasia, and decreased expression of defense-associated genes, including DEFB35 in the lung [18,19]. Notably, the expression of Foxa2 is suppressed both in mouse models and in humans with allergic asthma [18–20]. Type 2 cytokines, such as IL 4, IL 5, and IL 13, can significantly suppress Foxa2 expression [18,19]. In addition, IL4 and IL13 can reduce the mRNA expression level of some DEFBs. [6]. Based on these studies, we hypothesized that Foxa2, expressed in the respiratory epithelial cells, may play a role in the pulmonary defense against bacterial infections through the regulation of DEFB1 in Th2-dominated airway inflammation. Hence, this study was conducted to investigate the role of Foxa2 in the pulmonary defense of Th2-dominated airway inflammation in mice and to elucidate the possible underlying mechanisms of DEFB1 regulation.

## Methods

### Cells, bacteria and reagents

The human bronchial epithelial cell line HBE135-E6E7 and *E*. *coli* (ATCC 25922) were obtained from American Type Culture Collection (Manassas, VA, USA). Recombinant human interleukin-13 (IL13) and ovalbumin (OVA, grade IV) were purchased from Sigma-Aldrich (St. Louis, MO, USA). The primary antibody against Foxa2 was purchased from Cell Signaling Technology (Danvers, MA, USA). Imject^™^ Alum Adjuvant (Al(OH)3-Mg(OH)2) was obtained from Thermo Scientific (Waltham, MA, USA). The Dual-Luciferase® Reporter Assay System (E1910) was obtained from Promega (Madison, Wisconsin, USA).

### Animals

Wild-type BALB/c female mice aged 8 to 12 weeks (weight: 20–22 g) were used for the allergic asthma and infection experiments. *Foxa2*^*loxP/loxP*^ mice with a BALB/c genetic background were kindly provided by Dr. Klaus Kaestner at the University of Pennsylvania (Philadelphia, PA). Foxa2 was conditionally deleted (*Foxa2*^*△/△*^) in the airway epithelial cells of mice aged 8 to 12 weeks using the SPC-rtTA-tet(o)7-CRE system as described in a previous study[21]. Mice were maintained under pathogen-free conditions and had free access to water and food without OVA until the experiments. Mice were anesthetized with an intraperitoneal injection of pentobarbital and euthanized with lethal dose of pentobarbital. The study was approved by the Institutional Animal Care and Use Committee of West China Hospital of Sichuan University(no.2018006A).

### Induction of allergic airway inflammation

Wild-type mice were randomly divided into four groups, and mice used for the allergic asthma model were sensitized by two intraperitoneal injections of either 2.25 mg Imject Alum or 20 μg OVA and 2.25 mg Imject Alum in 200 μl PBS on days 0 and 14. Allergic asthma mice were then sensitized on days 21, 22, and 23 by inhalation of an aerosol of OVA within 50 minutes of generation by nebulization (PARI, Starnberg, Germany) in 1% OVA solution prepared in PBS(control mice received aerosol challenges of PBS at the same time).

### Infection of mice

*E*. *coli* was freshly subcultured from a -80°C stock and incubated on Luria-Bertani (LB) agar plates twice before each experiment. The bacterial count was measured in 1 ml of PBS by OD(600 nm) and confirmed by serial dilution and subculturing in LB Agar medium; colony-forming units (CFUs) were counted after 24 hours of culturing at 37°C. OVA-sensitized mice (on day 24), *Foxa2*^*△/△*^mice (aged 4–8 weeks) and control mice were all infected by intratracheal instillation of 50 μL of PBS containing *E*. *coli* of 1×10^6^ CFU/ml as described previously[22]. After twenty-four hours’ infection, mice were sacrificed, and lung and bronchoalveolar lavage fluid (BALF) lavaged three times using 1 ml of PBS were harvested as described elsewhere[22]. The cells in BALF were counted with a hemocytometer, and 100 μL cell suspensions were stained by Wright-Giemsa staining. The bacterial count was normalized to 1 mg of lung tissue homogenate and was measured by the serial dilution method as described above.

### Quantitative real-time PCR

Lung tissue homogenate and cell mRNA were extracted with TRIzol reagent (Invitrogen, Carlsbad, CA). cDNA was synthesized from 500 ng of RNA with a ReverTra Ace qPCR RT Kit (TOYOBO, Japan) and subjected to quantitative real-time PCR using specific mRNA primers targeting Foxa2, DEFB1, IL 4, IL 13, TNF α, IL 1β, Muc5ac, β-actin and GADPH (S2 File). For each sample, the level of gene expression was normalized to its own β-actin mRNA or GADPH mRNA.

### Measurement of DEFB 1 and IgE protein levels

Levels of DEFB 1 in lung tissue homogenates and BALF- and OVA-specific serum IgE were measured by enzyme-linked immunosorbent assays (ELISAs) (DEFbeta1 ELISA kit, BioSource, San Diego, USA; Mouse OVA-sIgE ELISA Kit, ALHAMBRA, CA, USA) according to the manufacturers’ instructions. All samples were tested in duplicate and read at 450 nm. The lung tissue homogenate was normalized to 1 mg. The low limit of detection for each assay was 12.5 pg/mL and 2.8 ng/mL for DEFB 1 and IgE, respectively. Standard curves were generated for every plate, and the average zero standard optical densities were subtracted from the rest of the standards, controls, and samples.

### RNA interference and expression of Foxa2 in HBE

siNC (targeting no genes in mouse, rat, and human) and siFoxa2 (catalog: si-h-FOXA2_005, targeting Foxa2, target sequence: 5'-GGGATGAACGGCATGAACA-3') were synthesized by Guangzhou Ribobio (Guangzhou, China). The ribo FECT^™^ CP Transfection Kit was obtained from Ribobio for transfection of siRNA according to the manufacturers’ instructions. The Foxa2-expressing vector (CMV-MCS-IRES-EGFP-SV40-Neomycin), using the reference sequence NM_021784, was synthesized by GeneChem (Shanghai, China). Lipofectamine 3000 (Thermo Fisher Scientific, USA) was -used for transfection according to the manufacturers’ instructions. HBE cells were plated onto 12-well, flat-bottom tissue culture plates (Corning Incorporated, USA) at a density of 1 × 10^5^ cells/well in DMEM high-glucose medium (Gibco, Thermo Fisher Scientific, USA). Cells were plated to reach 80–90% confluence on the day of transfection. Cells were allowed to recover in DMEM for 48 hours after transfection.

### Western blotting for expressional analysis

Western blot analysis was performed on lung homogenates from *Foxa2*^*Δ/Δ*^ mice and control littermates. The protein was separated by 10% SDS-PAGE, transferred, and immobilized on nitrocellulose membranes. Membranes were incubated with rabbit anti mouse Foxa2 ((1:1000; Cell Signaling Technology, Danvers, MA, USA) at 4°C overnight followed by 3–4 washes and incubated with HRP conjugated secondary antibodies obtained from zsbio (Beijing, China).

### Reporter plasmid construction and dual luciferase assays

We cloned the promoter–containing DEFB1-1099 luciferase reporter construct (starting from the ATG translation), by referring to [23], into the MCS (multiple cloning sites) of the pGL3-Basic reporter vector and named the resulting construct pGL3-DEFB1. The luciferase activity of pGL3-DEFB 1 was tested in 293T cells (S1 Fig). HBE cells were seeded in 12-well plates at a density of 1 × 10^5^ cells per well at 24 hours before transfection. The cells were transfected using Lipofectamine 3000 according to the manufacturer’s instructions. HBE cells were transfected with pGL3-Basic or pGL3-DEFB1 or pGL3-DEFB1 and pCMV-Foxa2 as well as pRL-TK. Dual luciferase assays were performed 48 hours after transfection following the manufacturer’s instructions. Normalized firefly luciferase activity (firefly luciferase activity/renilla luciferase activity) for pGL3-DEFB1 and pCMV-Foxa2 was compared to that of pGL3-Basic or pGL3-DEFB1, respectively. For each transfection, luciferase activity was averaged from four replicates.

### Histology

The lungs were perfused through the pulmonary artery with saline, then the lungs were fixed in 10% neutral formalin and desiccated and embedded in paraffin. Five-micrometer-thick tissue sections were processed for histological examination. Lung sections were stained with hematoxylin and eosin (HE) stain and periodic acid–Schiff (PAS) stain to visualize histopathological changes and mucus secretion. Levels of DEFB1 in the airway epithelium were quantified by immunohistochemistry (IHC).

### Data analysis

Data are presented as the mean±standard deviation. Statistical significance was evaluated using an unpaired t-test or one-way ANOVA, as appropriate. The results were considered statistically significant at p < 0.05.

## Results

### OVA sensitization increases lung inflammatory response

**Fig 1:**
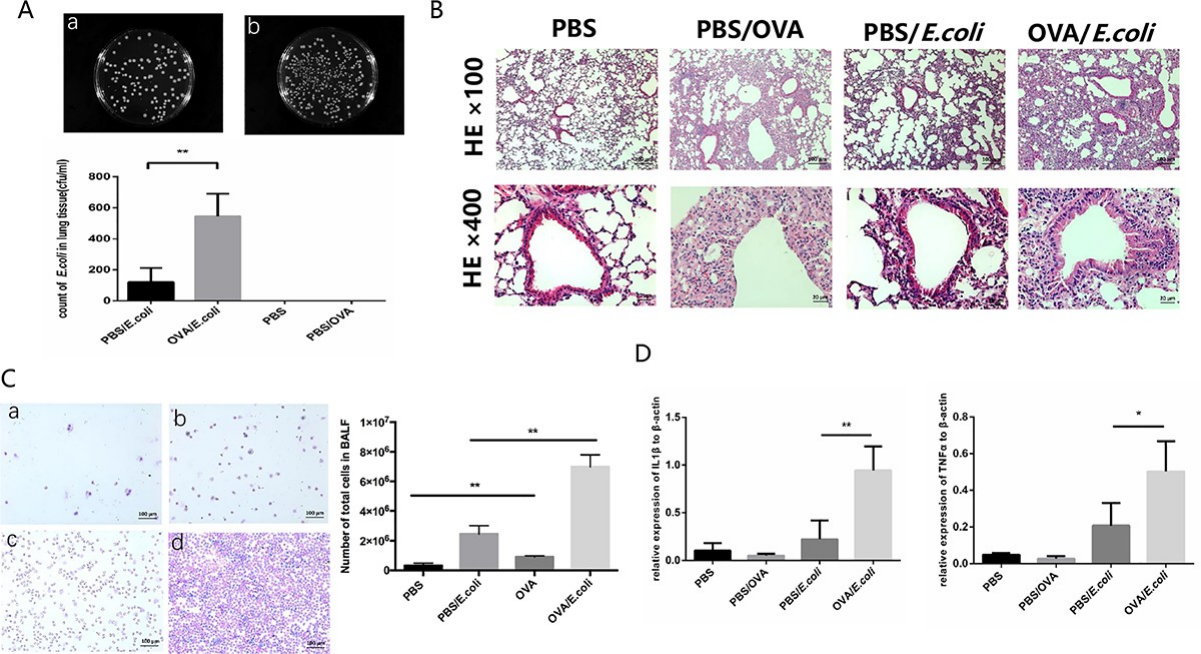
Antibacterial host defense is decreased in OVA-sensitized mice. A. The number of viable bacteria recovered from sensitized (OVA/*E*.*coli*) animals was significantly increased compared with the control animals (PBS/*E*. *coli*) after 24 hours’ infection. In all duplicate experiments, similar results were obtained. B. Representative HE staining of lung tissue sections. C. Representative Wright-Giemsa staining and total cell count of the BALF. D. mRNA expression levels of inflammatory cytokines IL-1β and TNFα.* indicates a significant difference at p < 0.05, ** indicates a significant difference at p < 0.01.

The extent of lung inflammation was evaluated by the bacterial count in lung tissues, HE staining of lung tissue sections, Wright-Giemsa staining and total cell count of the BALF and mRNA expression levels of inflammatory cytokines IL-1 β and TNFα. The mice were assigned to four groups (4–6 mice in each group) and were treated with: Imject Alum intraperitoneal injection and aerosol of PBS (PBS); OVA and Imject Alum intraperitoneal injection and aerosol of OVA (PBS/OVA); *E*. *coli* intratracheal instillation (PBS/*E*. *coli*); OVA and Imject Alum intraperitoneal injection and aerosol of OVA followed by *E*. *coli* intratracheal instillation (OVA/*E*. *coli*). Clearance of *E*. *coli* significantly decreased in lung tissue in OVA-sensitized mice (p < 0.05, n = 4, Fig 1A). Notably, no bacteria were recovered from the lung tissue homogenates of PBS or PBS/OVA mice. Histopathology showed eosinophils were the predominant infiltrating inflammatory cells in OVA-sensitized mice and neutrophils in PBS/*E*. *coli* and OVA/*E*. *coli* mice. A higher number of infiltrating cells were present in the OVA-sensitized and *E*. *coli* intratracheal instillation mice than in the control group mice (Fig 1B). In addition, there were more inflammatory cells in the BALF in the OVA-sensitized and *E*. *coli* intratracheal instillation mice than in the control group mice (p < 0.05, n = 4, Fig 1C). The expression of IL1β and TNFα mRNA in the lungs increased in OVA/*E*. *coli* mice compared to the control mice (p < 0.05, n = 4, Fig 1D). These results show heavy bacterial burdens accompanied by serious inflammatory responses in OVA/*E*. *coli* mice and suggest impaired airway defense against *E*. *coli* invasion in OVA-induced allergic responses.

### Decreased expression of DEFB1 in OVA-sensitized mice

**Fig 2:**
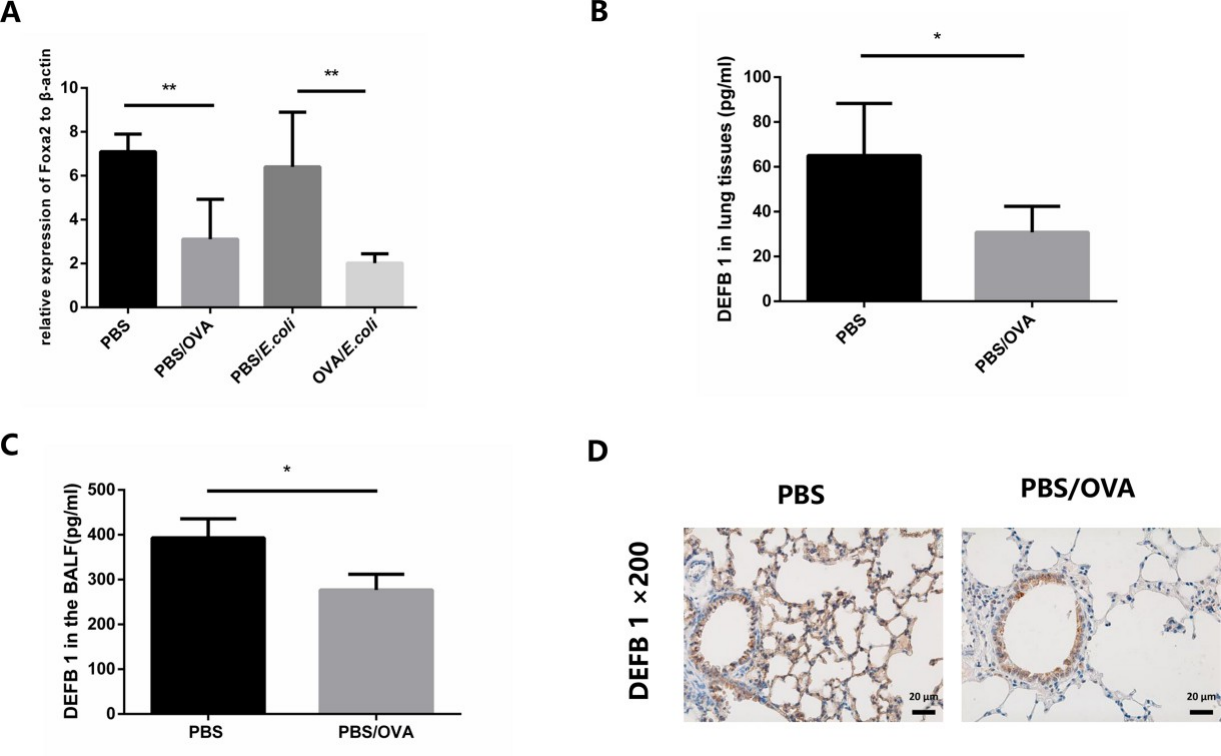
DEFB1 expression was decreased in OVA-sensitized mice. A. Relative expression levels of DEFB1 mRNA in four groups of mice. B. DEFB1 protein levels in lung tissue homogenates of OVA-sensitized and control mice measured by ELISA. C. DEFB1 protein levels in the BALF of OVA-sensitized mice and control mice measured by ELISA. D. Representative immunohistochemical staining of DEFB1 proteins (brown) in OVA-sensitized airway epithelial cells of OVA-sensitized and control mice. * indicates a significant difference at p < 0.05, ** indicates a significant difference at p < 0.01.

Previous studies showed decreased expression of DEFB2 and DEFB35 when allergic inflammation was dominant [6,18]. To determine whether the expression of DEFB1 was reduced in OVA-sensitized mice, we carried out RT-PCR, ELISA experiments, and immune staining to evaluate the DEFB1 expression level in OVA-induced allergic mice. Fig 2A shows consistently lower expression levels of DEFB1 mRNA in PBS/OVA and OVA/*E*. *coli* mice than those in control mice. *E*. *coli* intratracheal instillation did not induce significant expression of DEFB1 (confirmed by S2 Fig), consistent with previous studies [13]. Proteins both in lung tissue homogenates and BALF of OVA-sensitized mice, the expression levels of DEFB1 were lower than those in control mice (p < 0.05) (DEFB1 was not induced by infection, S2 Fig). The result of immunohistochemical staining showed that DEFB1 protein (brown), expressed in respiratory epithelial cells, decreased after OVA-sensitized compared to control mice (Fig 2D).

### Decreased expression of Foxa2 in OVA-sensitized mice

**Fig 3:**
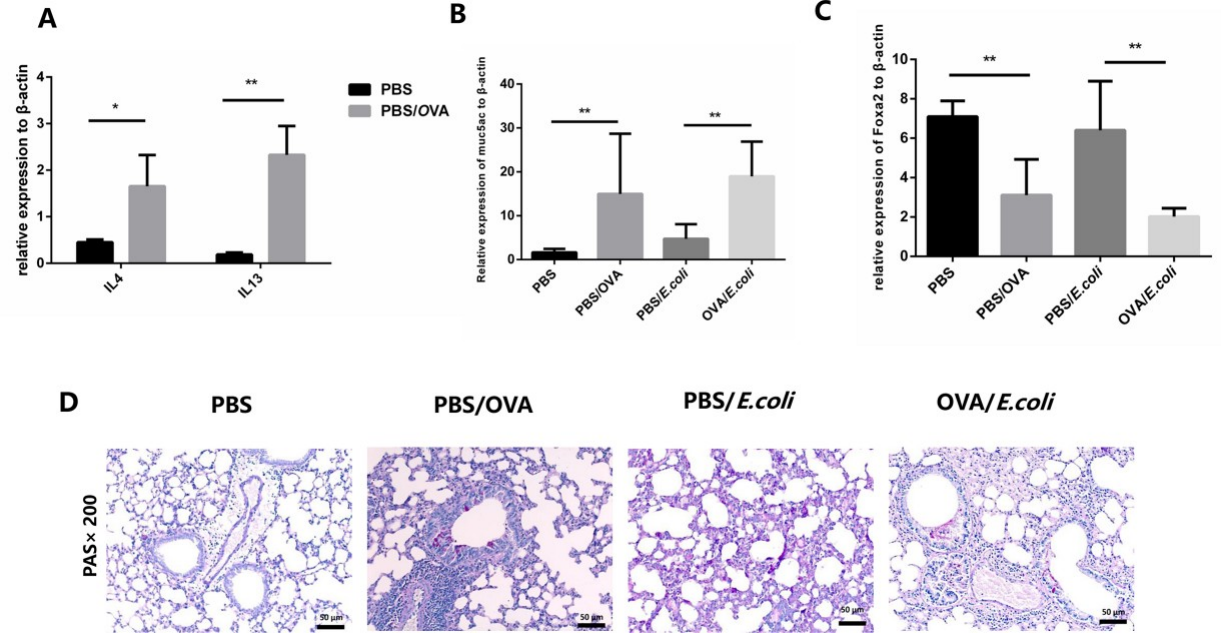
Foxa2 expression is decreased in OVA-sensitized mice. A. Relative expression of interleukin4 and interleukin13 MUC5AC in OVA-sensitized mice and those in controls. B. Relative expression of MUC5AC in four groups of mice. C. Relative expression levels of Foxa2 mRNA in four groups of mice. D. Representative PAS staining of lung tissue sections of four groups of mice. * indicates a significant difference at p < 0.05, ** indicates a significant difference at p < 0.01.

The relative mRNA expression of the Th2 cytokines IL 4 and IL13 and the secretion of MUC5AC and OVA-specific serum IgE that are characteristic of Th2-dominated allergic inflammation (Fig 3A, Fig 3B and S3 Fig) were in accordance with earlier studies [6,18]. The relative expression levels of IL4, IL13, and MUC5AC mRNA in OVA-sensitized mice were significantly higher than those in control mice (p < 0.05). Consistent with previous studies[19,20], the relative expression of Foxa2 mRNA was suppressed in Th2-dominated airway inflammation (p < 0.05, Fig 3C) charactered in OVA-sensitized mice. The representative PAS staining of lung tissue sections from four groups of mice, showed increased secretion of mucus in OVA-sensitized mice compared to control mice.

### Compromised airway defense in mice with Foxa2 deletion

**Fig 4:**
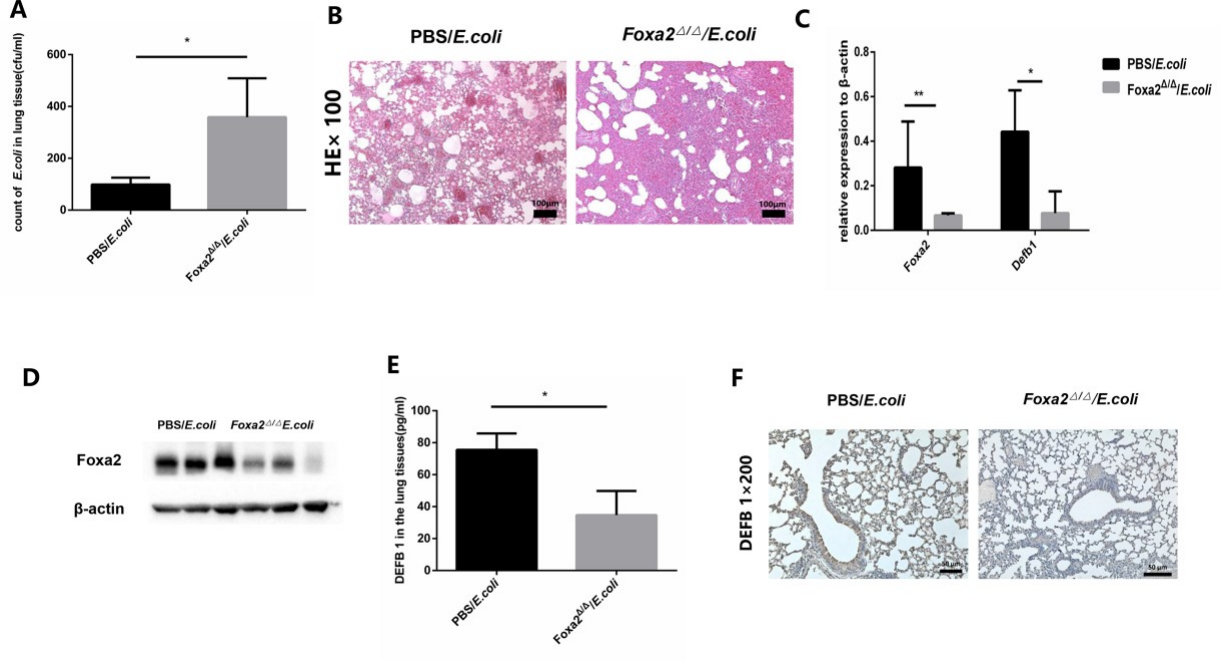
Host defense is compromised in mice with deletion of Foxa2 in respiratory epithelial cells. A. clearance of *E*. *coli* in *Foxa2*^*Δ/Δ*^ mouse lung tissue homogenate (p < 0.05, n = 3). In all duplicate experiments, similar results were obtained. B. representative HE staining of lung tissue sections from *Foxa2*^*Δ/Δ*^ mice and control mice. C.Relative expression levels of Foxa2 and DEFB1 mRNA in *Foxa2*^*Δ/Δ*^ mice (p < 0.05, n = 3). D. Foxa2 protein levels in *Foxa2*^*Δ/Δ*^ and control mice by western blot. E. DEFB1 protein levels in *Foxa2*^*Δ/Δ*^ mouse lung tissue homogenate measured by ELISA. F. Representative immunohistochemical staining of DEFB1 protein in OVA-sensitized airway epithelial cells infected with *E*. *coli* compared with epithelial cells of control mice. * indicates a significant difference at p < 0.05, ** indicates a significant difference at p < 0.01.

The expression of Foxa2 was shown to be suppressed by Th2 inflammation and was associated with the regulation of DEFB35 [18–20]. DEFBs are present in a β-defensin gene cluster on a single chromosome [10], and we hypothesized that Foxa2 might also regulate DEFB 1. We conditionally deleted Foxa2 in the respiratory epithelial cells of mice and evaluated the lung defense and expression of DEFB1. *Foxa2*^*Δ/Δ*^ mice showed spontaneous Th2-mediated inflammation in lung sections (S4 Fig). The clearance of *E*. *coli* in the lung tissue homogenate was lower in the *Foxa2*^*Δ/Δ*^ mice compared with control mice (p < 0.05. n = 3) (Fig 4A). The HE staining showed that enhanced lung inflammation was observed in *Foxa2*^*Δ/Δ*^ mice after *E*. *coli* exposure(Fig 4B). Levels of Foxa2 and DEFB1 mRNA and protein were measured in the lung tissues of *Foxa2*^*Δ/Δ*^ mice after *E*. *coli* exposure by RT-qPCR, western blot and immunohistochemical staining respectively. The results demonstrated that deletion of Foxa2 in respiratory epithelial cells decreased the expression of DEFB1 and Foxa2 (Fig 4C, 4D, 4E and 4F). Deletion of Foxa2 in the respiratory epithelial cells was associated to decrease expression of DEFB1.

### Foxa2 is associated with transcriptional regulation of DEFB1

**Fig 5:**
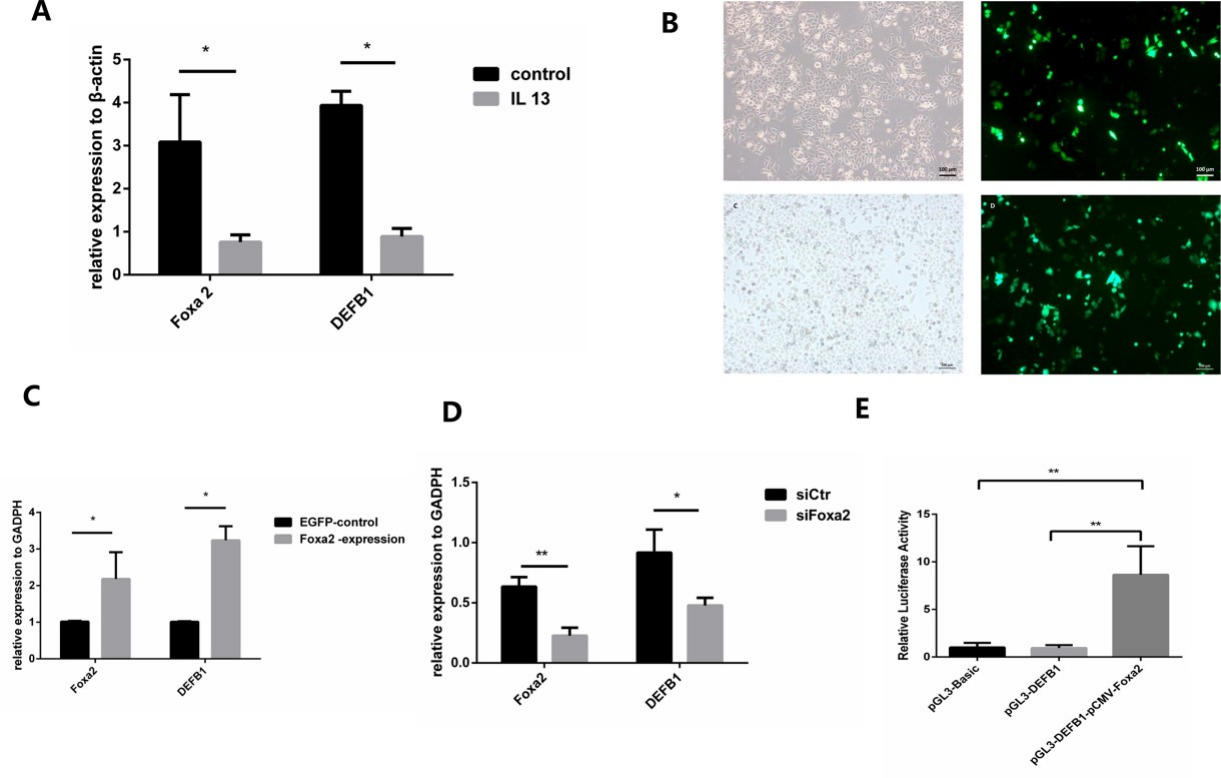
Regulation of DEFB1 by interference with the expression of Foxa2. A. IL13 suppresses the expression of Foxa2 mRNA and DEFB1 mRNA in HBE cells. B. Transfection of a Foxa2-expressing plasmid and control plasmid in HBE cells using light microscopy and fluorescence microscopy. C. Relative expression of Foxa2 mRNA and DEFB1 mRNA in Foxa2-expressing and control HBE cells. D. Relative expression of Foxa2 mRNA and DEFB1 mRNA in siFoxa2-transfected HBE and control cells. E. Relative luciferase activity of pGL3-Basic, pGL3-DEFB1, pGL3-DEFB1 and pCMV-Foxa2 (firefly luciferase activity/renilla luciferase activity). * indicates a significant difference at p < 0.05, ** indicates a significant difference at p < 0.01.

Due to consistent suppression of both Foxa2 and DEFB1 in Th2-dominated allergic inflammation, and association of Foxa2 with innate airway defense, we hypothesized that Foxa2 regulates DEFB1. IL13 was a representative cytokine in Th2-dominated allergic inflammation. To confirm the suppression of both Foxa2 and DEFB1 in Th2-dominated allergic inflammation, we detected the mRNA of Foxa2 and DEFB1 in HBE cells exposed to IL13. Fig 5A showed that a significant decrease in Foxa2 and DEFB1 mRNA in HBE cells after IL13 exposure. To seek mechanisms by which Foxa2 in expression of DEFB1, siRNA and expression plasmid were used to down-regulate or up-regulate Foxa2 expression in HBE cells. Foxa2 was significantly increased in HBE cells after pCMV-Foxa2 plasmid transfection (Fig 5C). Increased DEFB1 was also observed in HBE cells after up-regulation Foxa2 (Fig 5C). Meanwhile, we found that silencing Foxa2 expression leads to decreased expression of DEFB1(Fig 5D). In order to justify the role of Foxa2 in regulation of DEFB1. We conducted dual Luciferase reporter assay. It was found that up-regulation of Foxa2 expression significantly increased the activity of the DEFB1 promoter (Fig 5E). In conclusion, FOXA2 participates in the regulation of pulmonary defenses by affecting the transcription level of DEFB1.

## Discussion

Th2-dominated allergic diseases and asthma are global health problems causing severe illness and disabilities worldwide [1]. Epidemiological data show an increased prevalence of allergic diseases and asthma in recent years [1–3]. Observational studies identified the risk of infections in allergic diseases and asthma [3–6]. Elucidation of the molecular mechanisms of the airway defense against the invading microorganisms in Th2-dominated allergic diseases might provide a scientific basis for improving the care of this disease.

Foxa2 is highly conserved and widely expressed during murine embryogenesis and in adult tissues, where it functions as a primary transcription factor mediating the expression of genes involved in homeostasis and defenses, especially in the lung tissues [18]. Studies report decreased levels of Foxa2 in allergic diseases and asthma [20,24]. Therefore, genes associated with defenses may be affected by the depletion of Foxa2 in allergic diseases and asthma. Studies from both clinical investigations and animal experiments confirmed an increased risk of infections from allergic diseases [3–6,20].

Airway epithelial cells are the first line of defense against invading pathogens by secreting DEFBs and other antimicrobial compounds to protect the epithelial cells and the host [8]. DEFB1 proteins are important antimicrobial components that kill *E*. *coli* at micromolar concentrations *in vitro* [25]. DEFB1 is also an essential component of innate immunity and is secreted constitutively by the epithelial cells [11]. During infection, other types of DEFBs are induced by inflammatory factors [11], thus suggesting that DEFB1 may play a pivotal role in defense against invading pathogens when the host is confronted by a small number of microbes or in the early stages of an infection. Thus, DEFB1 depletion might enhance the probability of developing infections from an early stage. Previous studies suggested an association of an impaired activity or secretion of DEFB1 with decreased clearance of bacteria and increased risk of infections [9,12,25,26]. In contrast to CF in which DEFB1 is inactive [11], allergic asthma may suppress DEFB1 secretion and increase colonization by bacterial pathogens and the risk of infection. In our present study, reduced expression of Foxa2 downregulated DEFB1 and might have compromised bacterial clearance, leading to a more severe inflammatory response. Although Baines KJ et al. reported an elevated level of DEFB1 in COPD and severe asthma, due to the phenotype of asthma being indeterminate, bias may have arisen from asthma phenotypes other than allergic asthma, and the authors did not exclude the allergic condition in healthy controls and COPD populations [17].

Considering that innate immunity is critical for early protection against the overgrowth of pathogens, we used a short *E*. *coli* infection time model (24 hours). Immediate eradication of pathogens plays a crucial role in host defense against bacterial invasion. Innate immune responses are the primary defense mechanism against early pathogen invasion. Thus, understanding the precise airway defense mechanism offers a scientific basis for improving treatment strategies to decrease the risk of infection. Similar to earlier studies, *E*. *coli* infection did not upregulate the secretion of DEFB1 significantly [27,28]. Notably, β-defensins are multifunctional modulators. In addition to their innate antimicrobial activity and inherent immune component, β-defensins can also mediate the specific response of acquired immune responses [12].

Although DEFBs are found increasingly in different cells or organs, DEFB1 is expressed constitutively in the airway epithelial cells, whereas the induced expression of other defensins is seen during infections. This result showed the potential role of DEFB1 in preventing and reducing the risk of airway infection. Consistent with previous studies, we observed decreased expression of DEFB 2 in OVA/*E*. *coli* mice [S5 Fig]. DEFB molecules on mucosal surfaces may aggregate to eliminate pathogens during infection [12]. Further studies are needed to elucidate the regulatory mechanism of other DEFBs.

Our studies have several limitations. Although DEFB1 is an integral part of the innate immune defense system, other components should be investigated for their role in defenses against bacterial invasions, especially in allergic asthma. Different types of DEFBs or lysozymes may work together to defend against the potential pathogens [18]. In addition, the role of Foxa2 may not be limited to the regulation of DEFB1, and other aspects of pulmonary defense response to pathogen invasion, such as mucus secretion, may also be involved in the function of Foxa2 [18].

In summary, suppressing Foxa2 expression impairs pulmonary defenses and DEFB1 expression in mice. Foxa2 is involved in the transcriptional regulation of DEFB1, and downregulation of DEFB1 due to suppression of Foxa2 expression is likely to contribute to increased infection risk in the Th2-dominated airway inflammation.

## Supporting information

S1 File Raw_images of Western blotting. (PDF)

S2 File Table and methods. mRNA primers used in qPCR studies; Quantity of tissue sampling; Transgene Genotype.
(DOCX)

S1 Fig The luciferase activity of pGL3-DEFB 1 was tested in 293T cells. The luciferase activity of pGL3-DEFB1 was higher than that of pGL3-Basic, demonstrating the activity of DEFB 1 promoter sequence.
(TIF)

S2 Fig *E*. *coli* intratracheal instillation did not significantly induce the expression of DEFB1 (NS, P >0.05). (TIF)

S3 Fig OVA-specific serum IgE levels in OVA/*E*. *coli* and control measured by ELISA. (TIF)

S4 Fig Representative HE staining of lung tissue sections of *Foxa2*^*Δ/Δ*^ and control mice. (TIF)

S5 Fig Expression of DEFB2 in OVA/*E*. *coli* and control mice as measured by ELISA (DEFB2 ELISA kit, Jiangsu Meibiao Biological Technology Company Limited, Jiangsu, China). The levels of DEFB2 proteins were normalized in 50 mg of lung tissue.
(TIF)

S6 Fig Relative expression of Foxa2 in *Foxa2*^*Δ/Δ*^ and control mice(measured by Western blotting). (TIF)

## References

[1] Global Strategy for Asthma Management and Prevention. Global Initiative for Asthma. 2018 https://www.ginasthma.org (Accessed August 21th, 2019).

[2] Li J, Sun B, Huang Y, Lin X, Zhao D, Tan G, et al A multicentre study assessing the prevalence of sensitizations in patients with asthma and/or rhinitis in China. *Allergy*. 2009,64:1083–92. 10.1111/j.1398-9995.2009.01967.x 19210346

[3] Helby J, Nordestgaard BG, Benfield T, Bojesen SE. Asthma, other atopic conditions and risk of infections in 105 519 general population never and ever smokers. *J Intern Med*. 2017,282:254–267. 10.1111/joim.12635 28547823

[4] Almirall J, Bolíbar I, Serra-Prat M, Roig J, Hospital I, Carandell E, et al New evidence of risk factors for community-acquired pneumonia: a population-based study. *Eur Respir J*. 2008,31: 1274–84. 10.1183/09031936.00095807 18216057

[5] Colak Y, Afzal S, Nordestgaard BG, Lange P. Characteristics and Prognosis of Never-Smokers and Smokers with Asthma in the Copenhagen General Population Study. A Prospective Cohort Study. *Am J Respir Crit Care Med*. 2015,192: 172–81. 10.1164/rccm.201502-0302OC 25914942

[6] Beisswenger C, Kandler K, Hess C, Garn H, Felgentreff K, Wegmann M, et al Allergic airway inflammation inhibits pulmonary antibacterial host defense. *J Immunol*. 2006,177:1833–7. 10.4049/jimmunol.177.3.1833 16849494

[7] Kang CI, Rouse MS, Patel R, Kita H, Juhn YJ. Allergic airway inflammation and susceptibility to pneumococcal pneumonia in a murine model with real-time in vivo evaluation. *Clin Exp Immunol*. 2009, 156:552–61. 10.1111/j.1365-2249.2009.03925.x 19438610 PMC2691986

[8] Eisele NA, Anderson DM. Host Defense and the Airway Epithelium: Frontline Responses That Protect against Bacterial Invasion and Pneumonia. J Pathog. 2011, 2011:249802 10.4061/2011/249802 22567325 PMC3335569

[9] Giamarellos-Bourboulis EJ, Platzer M, Karagiannidis I, Kanni T, Nikolakis G, Ulrich J, et al High Copy Numbers of β-Defensin Cluster on 8p23.1, Confer Genetic Susceptibility, and Modulate the Physical Course of Hidradenitis Suppurativa/Acne Inversa. *J Invest Dermatol*. 2016,136:1592–1598. 10.1016/j.jid.2016.04.021 27164300

[10] Nurjadi D, Herrmann E, Hinderberger I, Zanger P. Impaired β-defensin expression in human skin links DEFB1 promoter polymorphisms with persistent Staphylococcus aureus nasal carriage. *J Infect Dis*. 2013,207:666–74. 10.1093/infdis/jis735 23204181

[11] Singh PK, Jia HP, Wiles K, Hesselberth J, Liu L, Conway BA, et al Production of beta-defensins by human airway epithelia. *Proc Natl Acad Sci USA*. 1998,95:14961–6. 10.1073/pnas.95.25.14961 9843998 PMC24558

[12] Tomalka J, Azodi E, Narra HP, Patel K, O'Neill S, Cardwell C, et al β-Defensin 1 plays a role in acute mucosal defense against Candida albicans. *J Immunol*. 2015,194:1788–95. 10.4049/jimmunol.1203239 25595775 PMC4323878

[13] Moser C, Weiner DJ, Lysenko E, Bals R, Weiser JN, Wilson JM. Wilson. beta-Defensin 1 contributes to pulmonary innate immunity in mice. *Infect*.*Immun*. 2002, 70: 3068–3072. 10.1128/IAI.70.6.3068-3072.2002 12010999 PMC127957

[14] Ryan LK, Wu J, Schwartz K, Yim S, Diamond G.β-Defensins Coordinate In Vivo to Inhibit Bacterial Infections of the Trachea. Vaccines (Basel). 2018,6 pii: E57. 30154362 10.3390/vaccines6030057 PMC6161282

[15] Taggart CC, Greene CM, Smith SG, Levine RL, McCray PB Jr, O'Neill S, McElvaney NG. Inactivation of human beta-defensins 2 and 3 by elastolytic cathepsins. *J Immunol*. 2003,171:931–7. 10.4049/jimmunol.171.2.931 12847264

[16] Leung TF, Li CY, Liu EK, Tang NL, Chan IH, Yung E, et al Asthma and atopy are associated with DEFB1 polymorphisms in Chinese children. *Genes Immun*. 2006,7:59–64. 10.1038/sj.gene.6364279 16435024

[17] Baines KJ, Wright TK, Simpson JL, McDonald VM, Wood LG, Parsons KS, et al Airway β-Defensin-1 Protein Is Elevated in COPD and Severe Asthma. Mediators Inflamm. 2015, 407271 10.1155/2015/407271 26568662 PMC4629049

[18] Wan H, Xu Y, Ikegami M, Stahlman MT, Kaestner KH, Ang SL, Whitsett JA. Foxa2 is required for transition to air breathing at birth. *Proc Natl Acad Sci USA*. 2004,101:14449–54. 10.1073/pnas.0404424101 15452354 PMC521955

[19] Chen G, Wan H, Luo F, Zhang L, Xu Y, Lewkowich I, et al Foxa2 programs Th2 cell-mediated innate immunity in the developing lung. *J Immunol*. 2010,184:6133–41. 10.4049/jimmunol.1000223 20483781

[20] Park SW, Verhaeghe C, Nguyenvu LT, Barbeau R, Eisley CJ, Nakagami Y, et al Distinct roles of FOXA2 and FOXA3 in allergic airway disease and asthma. *Am J Respir Crit Care Med*. 2009, 180:603–10. 10.1164/rccm.200811-1768OC 19628779 PMC2753788

[21] Tang X, Liu XJ, Tian C, Su Q, Lei Y, Wu Q, et al Foxa2 regulates leukotrienes to inhibit Th2-mediated pulmonary inflammation. *Am J Respir Cell Mol Biol*. 2013, 49:960–70. 10.1165/rcmb.2013-0122OC 23822876 PMC3931118

[22] Tang X, Sun L, Jin X, Chen Y, Zhu H, Liang Y, et al Runt-Related Transcription Factor 1 Regulates LPS-Induced Acute Lung Injury via NF-κB Signaling. *Am J Respir Cell Mol Biol*. 2017, 57:174–183. 10.1165/rcmb.2016-0319OC 28314106

[23] Peyrin-Biroulet L, Beisner J, Wang G, Nuding S, Oommen ST, Kelly D, et al Peroxisome proliferator-activated receptor gamma activation is required for maintenance of innate antimicrobial immunity in the colon. *Proc Natl Acad Sci U S A*. 2010,107(19):8772–7. 10.1073/pnas.0905745107 20421464 PMC2889363

[24] Choi W, Yang AX, Waltenburg MA. FOXA2 depletion leads to mucus hypersecretion in canine airways with respiratory diseases. *Cell Microbiol*. 2018, 16: e12957. 10.1111/cmi.12957 30221439

[25] Valore EV, Park CH, Quayle AJ, Wiles KR, McCray PB Jr, Ganz T. Human b-defensin-1: an antimicrobial peptide of urogenital tissues. *J*. *Clin*. *Invest*. 1998,101: 1633–42. 10.1172/JCI1861 9541493 PMC508744

[26] Zhao C, Wang I, Lehrer RI. Widespread expression of beta-defensin hBD-1 in human secretory glands and epithelial cells. *FEBS Lett*.1996,396:319–22. 10.1016/0014-5793(96)01123-4 8915011

[27] Kumar V, Everingham S, Hall C, Greer PA, Craig AW. Calpains promote neutrophil recruitment and bacterial clearance in an acute bacterial peritonitis model. *Eur J Immunol*. 2014,44:831–41. 10.1002/eji.201343757 24375267

[28] Morrison GM, Davidson DJ, Kilanowski FM, Borthwick DW, Crook K, Maxwell AI, et al Mouse beta defensin-1 is a functional homolog of human beta defensin-1. *Mamm Genome*. 1998, 9:453–7. 10.1007/s003359900795 9585433

