# Supplementary figures and images for: Molecular characterization of pulmonary defenses against bacterial invasion in allergic asthma: The role of Foxa2 in regulation of β-defensin 1
Source: PLoS One. 2019 Dec 27;14(12):e0226517. doi: 10.1371/journal.pone.0226517 (PMC6934329; doi:10.1371/journal.pone.0226517)

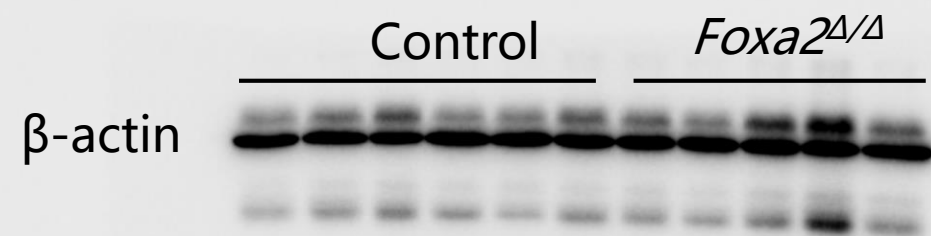

Foxa2

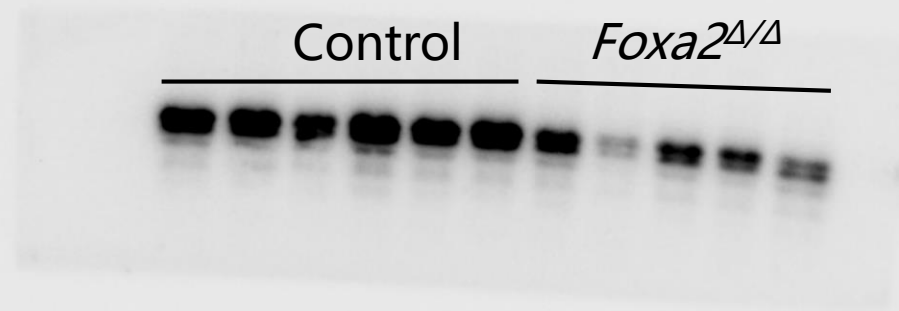

$\beta$ -actin

PBS/*E.coli*

*Foxa2* <sup>$\Delta/\Delta$</sup> /*E.coli*

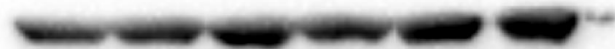

Foxa2

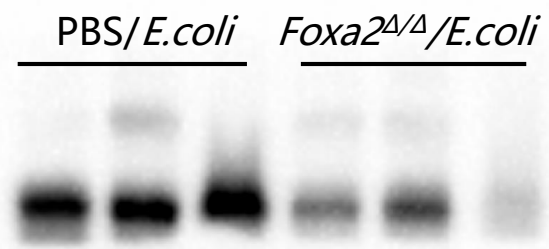

Supplement: S1 File — (PDF) [file pone.0226517.s001.pdf]

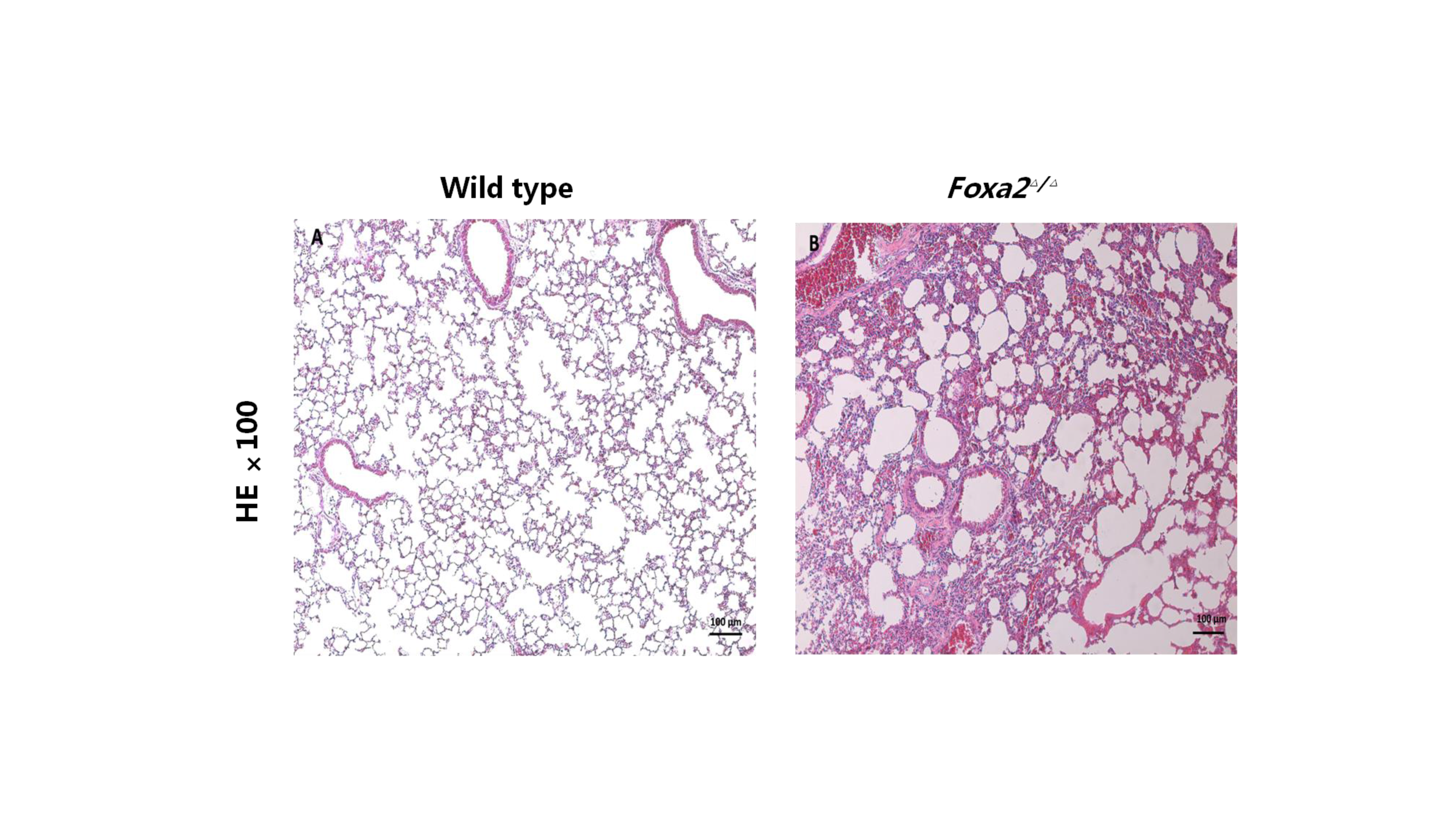

Supplement: S4 Fig — (TIF) [file pone.0226517.s006.tif]

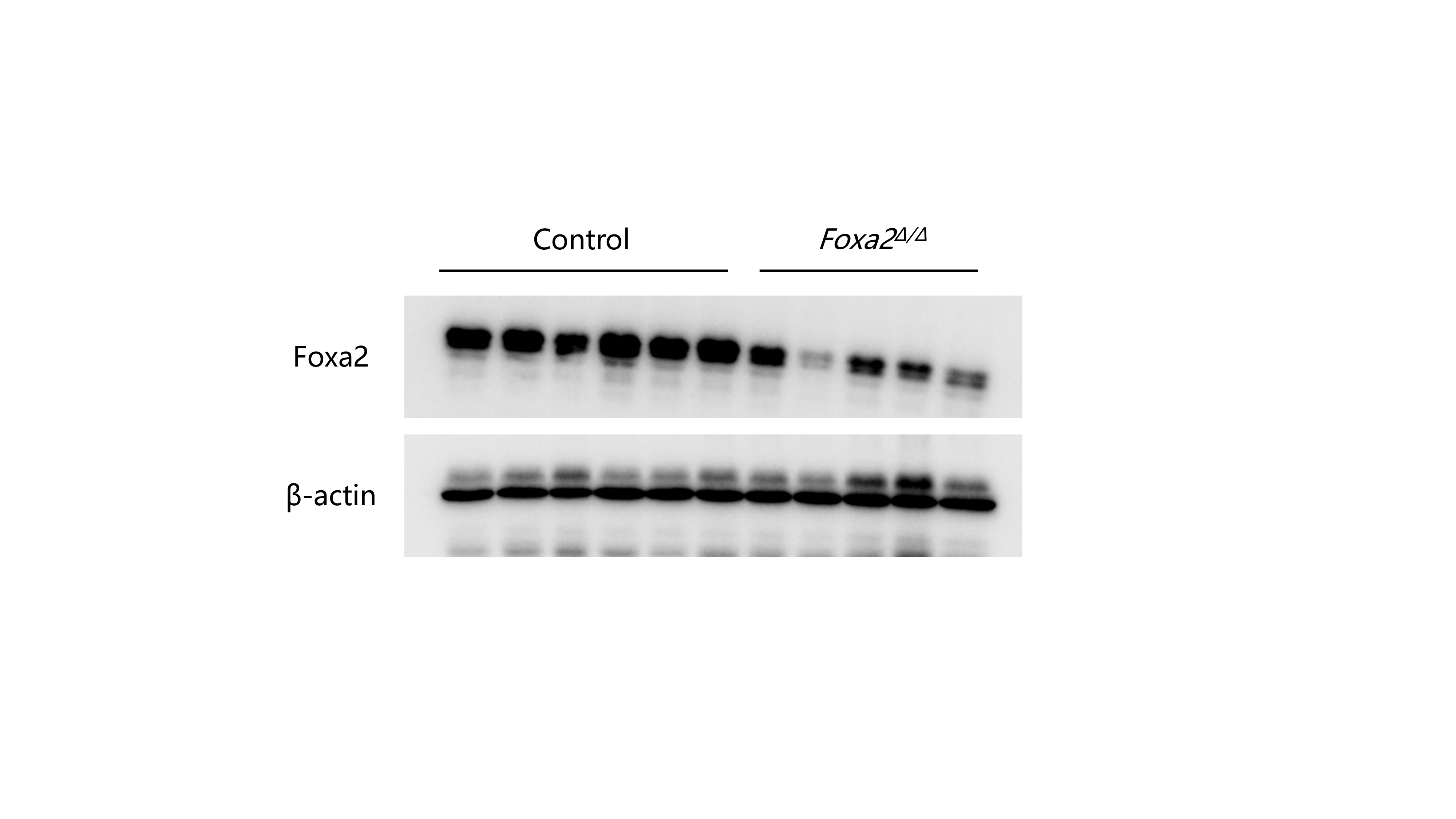

Supplement: S6 Fig — (TIF) [file pone.0226517.s008.tif]
