# Supplementary material for: Molecular characterization of pulmonary defenses against bacterial invasion in allergic asthma: The role of Foxa2 in regulation of β-defensin 1
Source: PLoS One. 2019 Dec 27;14(12):e0226517. doi: 10.1371/journal.pone.0226517 (PMC6934329; doi:10.1371/journal.pone.0226517)
Supplement: S2 File — mRNA primers used in qPCR studies; Quantity of tissue sampling; Transgene Genotype. (DOCX) [file pone.0226517.s002.docx]

S Table 1 mRNA primers used in qPCR studies.

| Primer sequence5'→3' | | | |
| --- | --- | --- | --- |
| gene | Forward primer | Reverse primer | Product length(bp) |
| HGAPDH | CCAAAAGGGTCATCATCTCT | TCTTGAGGCTGTTGTCATAC | 90bp |
| Hfoxa2 | GGAGCAGCTACTATGCAGAGC | CGTGTTCATGCCGTTCATCC | 83bp |
| Hdefb1 | AGATGGCCTCAGGTGGTAAC | CACTTGGCCTTCCCTCTGTA | 148bp |
| mβ-actin | ACTATCGGCAATGAGCGGTTC | ATGCCACAGGATTCCATACCC | 77bp |
| mfoxa2 | GTGGCCTAAGCGAGCTAAAG | TAATGGTGCTCGGGCTTCAG | 160bp |
| mdefb1 | ATGAAAACTCATTACTTTCTCCTGG | ACTACTGTCAGCTCTTACAACA | 217bp |
| mTLR4 | CGCTTTCACCTCTGCCTTCACTACAG | ACACTACCACAATAACCTTCCGGCTC | 109bp |
| mTLR2 | CTCCTGAAGCTGTTGCGTTAC | TACTTTACCCAGCTCGCTCACTAC | 123bp |
| mTNF-α | AGGAGGGAGAACAGAAACTC | GTGAGTGAAAGGGACAGAAC | 90bp |
| mIL-1β | GTACATCAGCACCTCACAAG | CAGTCCAGCCCATACTTTAG | 116bp |
| mMuc5ac | ACAGCATCTCCATGTACTCC | GTAGCTGAAGGTCTGACTGG | 131bp |
| mIL-4 | AGACTCTTTCGGGCTTTTCG | TGATGCTCTTTAGGCTTTCC | 106bp |
| mIL-13 | GAGGAGGGTTGAGGAGGAAG | TTTCTGTAGGGATGGGATGG | 136bp |

Supplemental method

Quantity of tissue sampling

The left lungs and tracheas from the same group of mice were fixed at least 8h in 4% paraformaldehyde and processed into paraffin blocks for immunohistochemistry, PAS and H&E staining. Lower lobe of right lung from each mouse was used for lung tissue homogenate in 1mL PBS. ELISAs and bacterial count were normalized by 1mg lung tissue.

Transgene Genotype

Foxa2loxP/loxP mice were generated at the University of Pennsylvania. Homologous recombination

between loxP sites was accomplished by using (tetO)7CMV-Cre mice, kindly provided by Corrinne Lobe. For lung-specific, doxycycline induced recombination, the SP-C-rtTA-/tg transgenic line was used. Triple-transgenic mice were generated by crossing(tetO)7Cre-/tgFoxa2loxP/loxP and SP-C-rtTA-/tg/Foxa2loxP/loxP. Littermates of all other genotypes served as controls. Transgenic mice were identified by PCR with genomic DNA from the tails of fetal and postnatal mice as described in ref. 18. The deletion efficiency of Foxa2 was justified by Western blotting as showed in S raw-Fig.

DEFB1 putative promoter region

UTR5’-3’ Insert length 1099

GGTAGAGTGGCTGAGGGTCACTCCACTCTGGGTGTCTCATGCCATGTGGCTGCAGTATGGGGTTCTCATTGGACCACCGTGACAGTGTAGGGTGGATGTCATTCCTGGAAACTGAGCCTCAAGCTGGGTTCTCCAGCAATGCCACCACCCCCCACCAACCCTGATATCCTCCGAATAAACTCTTCCCTTCCTTGGTCAGCGAGAGCCAGCGTCTGCTGCTTGCAAATAAGCCACTGAGACATCAGGAAAGCTGCTCACAGAATTACAATGAAAATCAGTAATAAATAAAATGTTTCTTAAATCTTTAGAATCAAGGAAAACCTGAGGGATACTTGGAGAGTCGCTAACAGCACCCCAATCTGGCAGTTGTAGCTCTGCAGGGTGGGCCCAAGCTGGCCCAGACCCTCACCCTCCAAGGGCCACACTGGGGGCTCACTTTCTGAGGAGTGCCCTTTGGAAACGTCCCAGGAACACGTCTAGTGGGAAAAGAGAAAAGTTGGTCCATCGAGGAGAGTGTTCTGCATAAGGGGAGAGATGAGAAGGTAGCCTTGGCCAGAGGAAGAAACTTCATTACAACCAGCTCTCCTTCTGCAAGGGAAGAGGGTGAAGTTTGAGTTTGTCTTGCAGGAAGACAATCAAACTAAAGAGGCCAACACCAGCTTAGAGCCGAGCGGCCCCCTGCTCAGAGCTTCCCTGTGGCTCTCCTCCATGTGATCCAGAAGGAGGGACTCCAGTGTGAACTGCCTGTTCCAGAAACCCCATCAGAACTGCCTAACCTAGAAAACCAAACAGGAGGAGCTGGCACCAGGGCTCCAGGCTGAAAGCTAAATCCAGCGGCAGCCAGATGGAGACAATGTGCCATGTGACTGCTGACTGCTCAGGGCAAATGACACCAGGGGTTAGCGATTAGAAGTTCACCCTTGACTGTGGCACCTCCCTTCAGTTCCGTCGACGAGGTTGTGCAATCCACCAGTCTTATAAATACAGTGACGCTCCAGCCTCTGGAAGCCTCTGTCAGCTCAGCCTCCAAAGGAGCCAGCGTCTCCCCAGTTCCTGAAATCCTGGGTGTTGCCTGCCAGTCGCCATGAGAACTTCCTAC

Red letters: Potential binding sites with the highest score of transcription factor Foxa2 predicted by Animal TFDB 3.0 website (P=4.6e-05). <http://bioinfo.life.hust.edu.cn/AnimalTFDB/>
